# Two Cases of Hemophagocytic Lymphohistiocytosis Associated with Disseminated Histoplasmosis Presented with Transient Pancytopenia

**DOI:** 10.1155/2022/9521128

**Published:** 2022-12-28

**Authors:** Novi Apriany, Usi Sukorini, Tri Ratnaningsih, Rizka Humardewayanti Asdie, Yanri Wijayanti Subronto, Susanna Hilda Hutajulu, Ibnu Purwanto, Mardiah Suci Hardianti

**Affiliations:** ^1^Study Program of Subspecialty, Department of Internal Medicine, Faculty of Medicine, Public Health and Nursing, Universitas Gadjah Mada/Dr. Sardjito General Hospital, Yogyakarta, Indonesia; ^2^Department of Clinical Pathology, Faculty of Medicine, Public Health and Nursing, Universitas Gadjah Mada/Dr. Sardjito General Hospital, Yogyakarta, Indonesia; ^3^Division of Tropical Medicine and Infectious Disease, Department of Internal Medicine, Faculty of Medicine, Public Health and Nursing, Universitas Gadjah Mada/Dr. Sardjito General Hospital, Yogyakarta, Indonesia; ^4^Division of Hematology and Medical Oncology, Department of Internal Medicine, Faculty of Medicine, Public Health and Nursing, Universitas Gadjah Mada/Dr. Sardjito General Hospital, Yogyakarta, Indonesia

## Abstract

Transient pancytopenia due to reactive bone marrow suppression often occurs in hemophagocytic lymphohistiocytosis (HLH), a syndrome resulting from excessive immune activation following a severe infection. We reported two cases with pancytopenia and disseminated histoplasmosis accompanied by HLH, initially suspected to be blood malignancies. Our first case documented the relevance between the improvement of pancytopenia and the clearance of Histoplasma capsulatum in serial bone marrow aspirations. The second case showed immense Histoplasma engulfment by the macrophage in relation to a severe clinical condition, followed by improvement of clinical symptoms in accordance with the recovery of pancytopenia. These two cases highlighted the importance of comprehensive and critical analysis for cases with concurrent pancytopenia and severe infection, since it may be that the pancytopenia underlies the severe infection or vice versa.

## 1. Case 1

**Figure 1:**
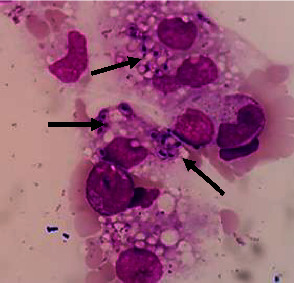
Initial bone marrow aspiration showing myelodysplasia syndrome (MDS) with refractory cytopenia with multilineage dysplasia (RCMD) subtype and infection with H. capsulatum inside the macrophages (taken 2 weeks after admission and as the initial point to start antifungal treatment).

**Figure 2:**
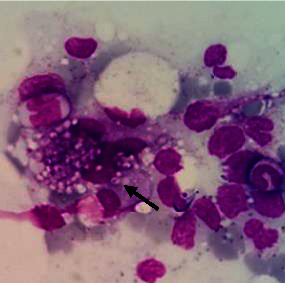
Bone marrow aspiration after 4 weeks of antifungal treatment showed the reduction of intracellular budding yeast of Histoplasma inside the macrophages.

**Figure 3:**
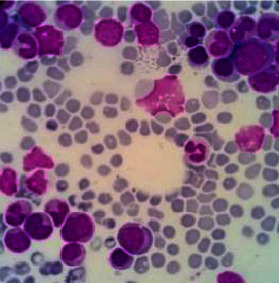
Bone marrow aspiration after 10 weeks of antifungal treatment showed macrophages without Histoplasma.

**Figure 4:**
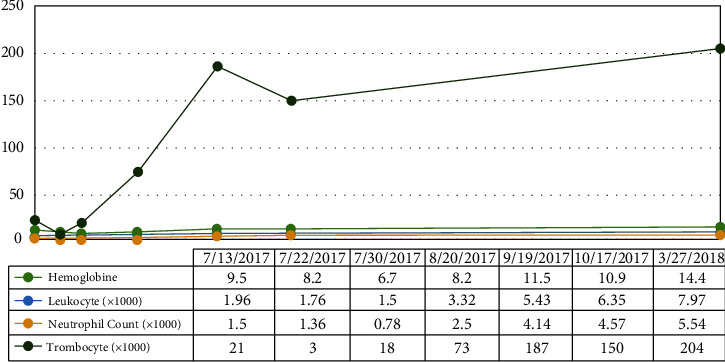
Improvement in peripheral blood parameters following treatment with an antifungal in case 1.

A 34-year-old woman was referred to our hospital with sepsis and prolonged fever. She had a productive cough for the last month. She had no previous history of any serious illness. She was a healthy person with an active life as a civil servant who travelled a lot in the country. Peripheral blood analysis showed pancytopenia with hemoglobin levels of 7.6 g/dl, a leukocyte count of 1.35 × 10^3^/*μ*l, a neutrophil count of 1.05 × 10^3^/*μ*l, and a platelet count of 12 × 10^3^/*μ*l. Procalcitonin was 15.69 ng/ml, albumin 1.85 g/dl, LDH 651 U/L, APTT 51.6 sec, and fibrinogen 0.62 g/L. She had negative results from any serological tests for HIV, hepatitis B, and C. Toxoplasma IgM, CMV IgM, and ANA IF were also negative. An abdominal computerized tomography (CT) showed hepatosplenomegaly, and a thorax X-ray showed bilateral pneumonia. She was initially managed with antibiotics, nutrition support, granulocyte colony-stimulating factor (G-CSF) injection, transfusion of packed red cells and platelets, albumin infusion, and symptomatic drugs to alleviate her clinical condition. The bone marrow aspiration (BMA) was performed in the second week, after a stable clinical condition. The result was a myelodysplasia syndrome (MDS) with a refractory cytopenia multilineage dysplasia (RCMD) subtype and infection with Histoplasma capsulatum (Figure 1). She was given voriconazole 200 mg intravenously every 12 hours for the first 3 days. Itraconazole at a dose of 3 × 200 mg was given on days 4–6 followed by 2 × 200 mg planned for 3 months. The sequential BMA revealed a gradual loss leading to complete clearance of H. capsulatum within ten weeks of treatment (Figures 2 and 3). We started to observe a gradual improvement in pancytopenia and the clinical condition after the commencement of the antifungal. The blood evaluation during treatment is shown in Figure 4. She was discharged from the hospital after forty days in good condition, with a hemoglobin level of 8.2 g/dl, a leukocyte count of 3.2 × 103/*μ*l, a neutrophil count of 2.5 × 103/*μ*l, and a platelet count of 73 × 103/*μ*l. The peripheral blood profile continued to improve and returned to normal during visits to the outpatient clinic. She regained her active life when visiting our outpatient clinic five months after the last bone marrow assessment free of Histoplasma.

## 2. Case 2

**Figure 5:**
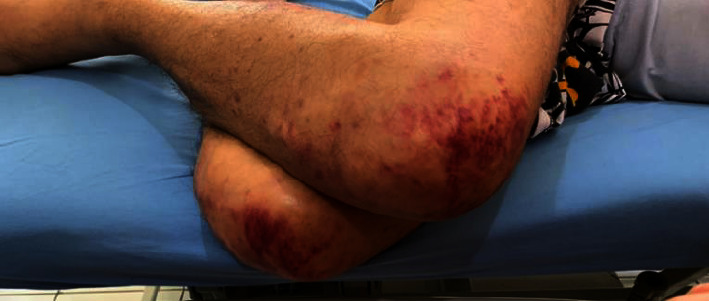
Large ecchymosis in the lower extremities.

A 29-year-old male was referred to our hospital due to general weakness for the last 7 months, abdominal discomfort, intermittent fever, and pancytopenia. According to his anamnesis, he was previously a healthy person without any serious illnesses. He was a computer technician and a tourist guide. He was suspected to have a blood malignancy by the previous referrer hospital due to repeated infections. He presented with several large ecchymoses in his lower extremities and trunk (Figure 5). An abdominal palpation revealed an enlarged liver measuring 3 cm below the costal margin and splenomegaly with Schuffner grade 2. The laboratory results revealed pancytopenia with a hemoglobin level of 6.2 g/dl, a leukocyte count of 1 × 10^3^/*μ*l, a neutrophil count of 0.69 × 10^3^/*μ*l, and a platelet count of 21 × 10^3^/*μ*l. Procalcitonin was 2.11 ng/ml, SGOT 107 U/L, SGPT 33 U/L, total bilirubin 4.96 mg/dl with direct bilirubin 4.8 mg/dl. There was a disseminated intravascular coagulation (DIC) as indicated with fibrinogen 0.35 g/L, APTT > 400 sec, PPT > 320 sec, and D-Dimer 572 ng/mL. The serology of HIV, hepatitis B and C, and ANA IF were negative. The thorax X-ray showed bronchitis.

**Figure 6:**
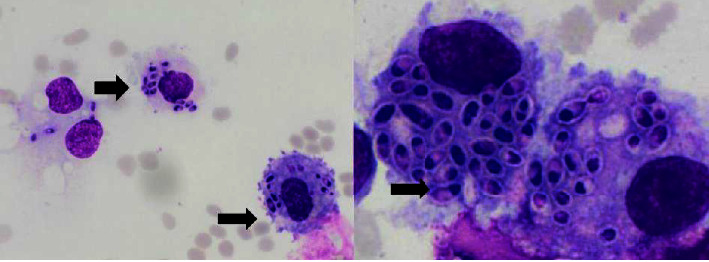
Bone marrow aspiration at the time of diagnosis showed Histoplasma capsulatum yeasts inside the macrophages.

**Figure 7:**
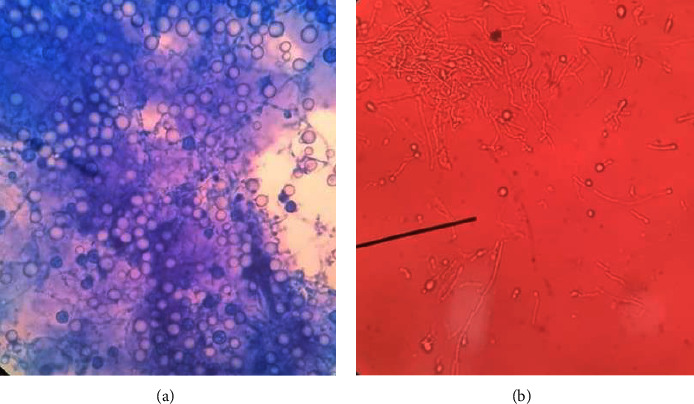
Histoplasma capsulatum yeast in methylene blue (a) and KOH (b) staining taken 5 weeks after starting BMA culture in Sabouraud agar.

**Figure 8:**
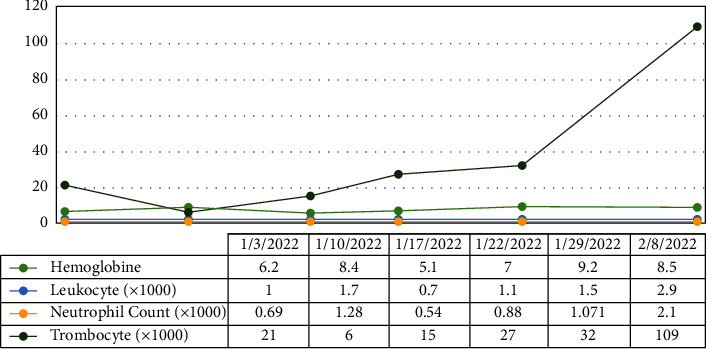
Improvement in peripheral blood parameters following treatment with an antifungal in case 2.

The BMA smear showed hemophagocytic lymphohistiocytosis (HLH) with abundant H. capsulatum inside the macrophage cells with dyserythropoiesis and dysgranulopoiesis (Figure 6). Sabouraud agar was used to grow some of the BMA samples. The isolate was subsequently taken after 5 weeks and stained with methylene blue and KOH (Figure 7). A sensitivity test showed good response to several antifungals, such as voriconazole, fluconazole, ketoconazole, and miconazole. The patient was managed with voriconazole 200 mg intravenously every 12 hours for 3 days, followed by itraconazole 3 × 200 mg in the first 3 days, followed by 2 × 200 mg planned for 3 months. Other therapies included intravenous antibiotics, G-CSF injection, supportive transfusion of packed red cells, platelets, fresh frozen plasma, and albumin infusion. We observed gradual improvement in clinical and laboratory parameters during hospitalization, and the patient was discharged after 30 days. The blood evaluation during treatment is shown in Figure 8. One week after discharge, a blood evaluation showed improvement in hemoglobin level of 8.5 g/dl, leukocyte count of 2.9 × 10^3^/*μ*l, neutrophil count of 2.1 × 10^3^/*μ*l, and platelet of count 109 × 10^3^/*μ*l.

## 3. Discussion

Histoplasma capsulatum is a dimorphic fungus that primarily causes pulmonary disease. The environmental reservoir of H. capsulatum is soil. There are two varieties that are pathogenic to humans, namely, H. capsulatum var. capsulatum and H. capsulatum var. duboisii [1]. Forms of mycelium (H. capsulatum) are found in the soil, especially in areas contaminated with bird or bat droppings [2]. After a detailed anamnesis with the 2 patients, the first patient reported that her house was not far from a big poultry farm, while the second patient had some birds as pet in his backyard. Such facts may be related to the repeated infections in the two cases.

There is a broad range of clinical presentations for histoplasmosis, from asymptomatic to symptomatic to a severe disseminated state. Disease signs and symptoms are mediated by the host's immune status. Other factors that play roles in the severity of the disease include the virulence of the fungal strain and the amount of inhaled inoculum [3, 4]. Infection in immunocompetent patients required a large quantity of inhaled H. capsulatum or exposure to a highly virulent strain of H. capsulatum [5]. These 2 presented cases were previously healthy individuals with no history of serious illness. Continuous exposure to a virulent strain accompanied by fluctuating immunity might have contributed to the development of severe infection in these cases.

Symptomatic clinical presentations include acute pulmonary disease, disseminated disease, and chronic pulmonary histoplasmosis. Symptoms of the disseminated disease include fever, weight loss, and respiratory complaints. Clinical findings may include lymphadenopathy, hepatomegaly, and splenomegaly, together with bone marrow toxicity on laboratory evaluation [3]. Our two cases presented the severe clinical condition of disseminated histoplasmosis in previously immunocompetent patients, with the second case being more severe with splenomegaly, congestive hepatopathy, obstructive jaundice, frank hemorrhage, and DIC.

The mechanism by which histoplasmosis can cause pancytopenia may be explained by the hemophagocytic lymphohistiocytosis (HLH) phenomenon, a life-threatening hyperinflammatory syndrome classified into primary and secondary forms[6]. Secondary or acquired HLH may result from a malignant, infectious, or autoimmune stimulus in the absence of an identifiable underlying genetic trigger [7]. Hemophagocytic lymphohistiocytosis is characterized by uncontrolled activation of NK/CTL that provokes the release of large amounts of proinflammatory cytokines such as IFN-*γ*, TNF-*α*, GM-CSF, M-CSF, and IL-2, resulting in hyperstimulation and systemic infiltration by macrophages, which in turn phagocytose blood cells, mostly red blood cell precursors, and secrete other cytokines responsible for myelosuppression, endothelial damage with coagulopathy, tissue injury, and NK/CTL incessant activation (IL-1, IL-6, and TNF-*α*) [6, 8]. The cytokine storm causes vascular endothelium damage and myelosuppression, which, in turn, induces fatal bleeding, infection, and multiorgan failure [6]. These two cases were referred from other hospitals with pancytopenia and severe infection, which required a comprehensive and critical analysis to determine whether the pancytopenia underlies the infection or vice versa. The findings of disseminated Histoplasma infection in the BMA eventually led to the most possible explanation of HLH as the cause of pancytopenia in these cases.

The gold standard for the diagnosis of histoplasmosis involves either the recovery of H. capsulatum in culture from a clinical specimen or histopathological demonstration of the characteristic intracellular forms of H. capsulatum in infected tissues [9]. Both of the presented cases showed the pathogen in the BMA smears. The recovery of H. capsulatum from the culture of BMA in Sabouraud agar from the second case confirmed the diagnosis.

The diagnosis of HLH was based on clinical, laboratory, and histopathological findings. In adults, it is based on the HLH-2004 diagnostic criteria in conjunction with clinical judgment and the patient's history. The criteria included ≥ 5 of the 8 diagnostic criteria listed in Table 1 [10]. Both of our cases fulfilled 5 of the 8 diagnostic criteria for HLH in terms of fever, splenomegaly, cytopenia, hemophagocytosis in BMA, and hypofibrinogenemia.

Myelodysplastic syndromes (MDS), also called ineffective hematopoiesis, are indicated by bone marrow failure and a tendency to acute myeloid leukemia transformation. It is a heterogenous group of malignancies arising from distorted hematopoietic stem cell function, inflammatory, innate immune deregulation, and multiple genomic events. Myelodysplastic syndromes are usually suspected by the presence of cytopenia on a routine analysis of peripheral blood. This prompts the evaluation of bone marrow cell morphology (aspirate) and cellularity (biopsy). The BMA allows for a detailed evaluation of cellular morphology and blast percentage. The diagnosis of MDS is established by the presence of dysplasia [11, 12]. All causes of secondary bone marrow failure should be put on the list of different diagnoses for MDS, and one of them is infection [11]. Histoplasmosis infection can also cause disseminated histiocytosis. The bone marrow is hypocellular, with reduced erythropoiesis and granulopoiesis [13]. In the first case, the initial BMA smears were identified as MDS with an RCMD subtype and an infection with Histoplasma capsulatum. While in the second case, the BMA also showed an immense engulfment of Histoplasma by the macrophages as well as the dysplasia of the three lineages, which was similar to the MDS feature. It was not easy to judge whether the cytopenia was due to MDS or HLH caused by disseminated histoplasmosis. However, the improvement in the serial BMAs of case 1 indicated by diminishing Histoplasma along with normalization of the peripheral blood profile strongly supported a reactive bone marrow suppression, resulting in the picture of myelodysplasia at the time of diagnosis. Although we did not repeat the bone marrow aspiration after complete recovery of the peripheral blood profile in case 1, we assured the patient that the MDS was only transient or temporary.

The antifungal agents that have been proven to be effective and preferred for the treatment of histoplasmosis include amphotericin B, liposomal amphotericin B, amphotericin B lipid complex, and itraconazole. For patients with moderate to severe disseminated histoplasmosis, an amphotericin B preparation is recommended for a minimum of 1–2 weeks, followed by oral itraconazole at a dose of 200 mg twice daily for a minimum of 12 months. In patients with mild to moderate disseminated histoplasmosis, itraconazole 200 mg twice a day for at least 12 months is an alternative [14, 15]. Voriconazole and liposomal amphotericin B (AmBisome) were also shown to be similarly effective for empiric therapy. There were fewer severe infusion-related reactions related to voriconazole and less nephrotoxicity than with liposomal amphotericin B [16]. Voriconazole has been used in the treatment of pulmonary histoplasmosis and disseminated histoplasmosis and may improve the clinical outcome [17, 18]. The secondary hemophagocytic lymphohistiocytosis (HLH) syndrome, which follows a severe infection such as disseminated histoplasmosis, is a life-threatening hyperinflammatory syndrome that requires treatment of the inciting disease to be treated and controlled [19]. The use of voriconazole and itraconazole to eradicate H. capsulatum as the causative agent in our cases effectively reversed the clinical conditions in coherence with the improvement of laboratory parameters.

## 4. Conclusion

We have reported two cases of transient pancytopenia as a result of reactive bone marrow suppression related to secondary hemophagocytic lymphohistiocytosis (HLH) due to disseminated Histoplasma capsulatum infection. These two cases reminded the clinicians of the importance of comprehensive and critical analysis when finding a case of pancytopenia accompanied by severe infection. It may be the pancytopenia that underlies the severe infection, or vice versa. When the latest occurs, the eradication of the inciting agents will reverse the pancytopenia condition.

## Figures and Tables

**Table 1:** HLH-2004 diagnostic criteria.

The diagnosis of HLH can be established if criterion 1 or 2 is fulfilled
(1) A molecular diagnosis consistent with HLH
(2) Diagnostic criteria for HLH fulfilled (5 of the 8 below)
Fever
Splenomegaly
Cytopenias (affecting ≥ 2 of 3 lineages in the peripheral blood)
Hemoglobin < 90 g/L (hemoglobin < 100 g/L in infats < 4 weeks)
Platelets < 100 × 10^9^/L
Neutrophils < 1.0 × 10^9^/L
Hypertriglyceridemia and/or hypofibrinogenemia
Fasting triglycerides ≥ 3.0 mmol/L (ie, ≥265 mg/dL)
Fibrinogen ≤ 1.5 g/L
Hemophagocytosis in bone marrow or spleen or lymph nodes. No evidence of malignancy
Low or no NK cell activity (according to local laboratory reference)
Ferritin ≥ 500 *μ*g/L
sCD25 (ie, soluble IL-2 receptors) ≥2400 U/mL
La Rosée et al. [10]

## Data Availability

All supporting data are indeed included within the article.
